# The Kat in the HAT: The Histone Acetyl Transferase Kat6b (MYST4) Is Downregulated in Murine Macrophages in Response to LPS

**DOI:** 10.1155/2018/7852742

**Published:** 2018-06-06

**Authors:** Smita Shukla, Carly Levine, Roopa Payanur Sripathi, Genie Elson, Carol Susan Lutz, Samuel Joseph Leibovich

**Affiliations:** ^1^Department of Cell Biology & Molecular Medicine, Rutgers Biomedical & Health Sciences, New Jersey Medical School, 185 South Orange Avenue, Newark, NJ 07103, USA; ^2^The School of Graduate Studies, Rutgers Biomedical & Health Sciences, New Jersey Medical School, 185 South Orange Avenue, Newark, NJ 07103, USA; ^3^Department of Microbiology, Biochemistry & Molecular Genetics, Rutgers Biomedical & Health Sciences, New Jersey Medical School, 185 South Orange Avenue, Newark, NJ 07103, USA

## Abstract

Epigenetic modulators, including histone methylases, demethylases, and deacetylases, have been implicated previously in the regulation of classical and alternative macrophage activation pathways. In this study, we show that the histone acetyl transferase (HAT) Kat6B (MYST4) is strongly suppressed (>80%) in macrophages by lipopolysaccharide (LPS) (M1 activation), while Kat6A, its partner in the MOZ/MORF complex, is reciprocally upregulated. This pattern of expression is not altered by LPS together with the adenosine receptor agonist NECA (M2d activation). This is despite the observation that miR-487b, a putative regulator of Kat6B expression, is mildly stimulated by LPS, but strongly suppressed by LPS/NECA. Other members of the MYST family of HATs (Kat5, Kat7, and Kat8) are unaffected by LPS treatment. Using the pLightswitch 3′UTR reporter plasmid, the miR-487b binding site in the Kat6b 3′UTR was found to play a role in the LPS-mediated suppression of Kat6B expression, but other as-yet unidentified factors are also involved. As Kat6B is a HAT that has the potential to modulate gene expression by its effects on chromatin accessibility, we are continuing our studies into the potential roles of this epigenetic modulator in macrophage activation pathways.

## 1. Introduction

Macrophages are tissue resident phagocytic cells that play key roles in immune responses, tissue debridement, angiogenesis, and wound repair following injury. Macrophages take up residence in developing tissues during embryogenesis, and their presence is maintained throughout the life of the animal in the uninjured state by a slow rate of turnover [1–6]. In response to injury or infection, monocytes are recruited from the circulation by chemoattractants produced at the sites of injury, and these monocytes differentiate into macrophages [7–9]. Both resident and recruited macrophages respond to local environmental stimuli, which modify the gene expression profile of these cells to produce cytokines, growth, and angiogenic factors that mediate inflammatory and anti-inflammatory responses, angiogenesis, and tissue repair.

The particular phenotype adopted by macrophages in response to environmental stimuli depends upon a variety of factors. Macrophages can be “classically activated” to assume an inflammatory phenotype, characterized by the expression of cytokines such as TNF*α* and IL-12, nitric oxide (NO) (via the inducible NO synthase), and proteolytic enzymes. This phenotype has been dubbed “M1” and is induced, at least in vitro, by interferon-*γ* (IFN-*γ*) alone or in combination with endotoxin (lipopolysaccharide (LPS)) or other Toll-like receptor (TLR) agonists. An “alternatively activated” phenotype, canonically termed “M2,” induced by IL-4 and IL-13 exhibits an anti-inflammatory phenotype, characterized by low expression of inflammatory cytokines and elevated expression of anti-inflammatory cytokines including IL-10, IL-1R*α*, and TGF*β*, as well as arginase-1 (in mice), CD206, and CD163 [10, 11]. Recently, recommendations for the description of macrophage activation have been proposed. These proposals clearly recognize the extreme flexibility of macrophage gene expression in response to external cues and recognize that the simple use of the term “M2” is misleading. A set of standards encompassing macrophage source, definition of activators, and a consensus collection of markers to describe macrophage activation was proposed to provide a common framework for the broad diversity of macrophage phenotypic modulation in response to exogenous stimuli [12]. In addition, these proposals recognize that additional modes of activation resulting in modifications of macrophage gene expression profiles have been described that do not conform to the simplified M1/M2 paradigm. In our studies, we have defined a pathway of activation that switches macrophages from an M1 phenotype to an M2-like phenotype that we have previously termed “M2d”, which requires stimulation of TLRs 2, 4, 7, or 9, together with stimulation of adenosine A_2A_ and A_2B_ receptors (A_2A_R and A2BRs) [13–20]. This M2d phenotype is characterized by low expression of inflammatory cytokines, elevated expression of anti-inflammatory cytokines including IL-10, upregulated expression of A_2A_Rs and A_2B_Rs, and strongly upregulated expression of VEGF. The M2d pathway of activation is independent of IL-4 and IL-13 and does not depend upon either IL-10 or IL-6 stimulation [16].

In our studies of the signaling pathways involved in macrophage activation, we carried out a detailed profiling of microRNA (miRNA) expression by murine macrophages activated by LPS (M1 activation) and LPS together with the adenosine A_2_R agonist 5′-N-ethylcarboxamidoadenosine (NECA) (“M2d” activation). MiRNAs are single-stranded RNAs composed of 21–23 nucleotides. These RNAs may function as posttranscriptional regulators of gene expression by binding to the 3′ untranslated region (3′UTR) of mRNAs. Each miRNA contains a seeding region that plays a key role in target binding and repression of gene expression [21]. Repression of expression may occur at the translational level or by promoting mRNA degradation [22, 23]. Prior studies have examined the effects of LPS on miRNA expression in macrophages [24–33]. MiRNAs including miR-155, miR-146a, and miR-210 have previously been shown to be involved in regulating the expression of cytokines such as TNF*α*, IL-6, and IL-10 [34–38]. In the study presented here, we identified a limited subgroup of miRNAs that were found to be regulated in response to LPS/NECA in comparison to LPS alone (Table 1). We initially chose to examine the role of miR-487b, which we found to be upregulated by LPS, but strongly downregulated by the combination of LPS and NECA (M2d activation). Bioinformatic analysis indicated a limited group of genes with conserved 3′UTR binding sites for miR-487b, including the histone acetyl transferase (HAT) Kat6b (MYST4), which is the focus of this manuscript. Studies of other miRNAs differentially modulated by LPS/NECA are currently underway.

Given the potential importance of epigenetic modifications in the regulation of macrophage differentiation and activation, we examined the expression in macrophages of Kat6b and other members of the MYST family of genes in response to LPS (M1 activation) and LPS/NECA (M2d activation). Strong and sustained suppression of Kat6b was observed in response to LPS. In addition, MYST3 was reciprocally upregulated by LPS, while MYST1, MYST2, and MYST5 were only marginally affected. We also examined the role of miR-487b in regulating the expression of Kat6b (MYST4). Our results suggest that the miR-487b site in the 3′UTR of the Kat6b gene may play a role in regulating the expression of the Kat6b gene, but that other, as-yet unidentified pathways regulated by LPS also contribute to the LPS-mediated suppression of Kat6b gene expression.

## 2. Materials and Methods

### 2.1. Macrophage Isolation and Culture

Thioglycolate-induced peritoneal macrophages were prepared as previously described. Briefly, C57BL/6J male mice (8 weeks of age, Jackson Laboratories, Bar Harbor, Maine) were injected intraperitoneally (ip) with thioglycolate broth (3.5 ml). Four days later, mice were sacrificed by cervical dislocation, injected ip with 3.5 ml sterile PBS, and the peritoneal cavity exudate was then harvested. The cells were pelleted, washed (3x) with PBS, resuspended in RPMI 1640 medium (Sigma, St. Louis, MO) containing 10% heat-inactivated fetal bovine serum (FBS), (Atlanta Biologicals, Lawrenceville, GA), 1% Pen-Strep 100x Solution, and 2% L-glutamine 200 mM Solution (Sigma), and plated at a density of 8 × 10^6^ cells per 100 mm dish. The cells were incubated at 37°C in a 5% CO_2_ tissue culture incubator overnight. Nonadherent cells were removed by washing, and the medium was replaced with RPMI 1640-1% FBS. The adherent cells, consisting of >95% macrophages, were then treated as follows: (a) stimulated with 100 ng/ml *E. coli* LPS (TLR4 agonist, purified to be free of TLR2 agonists, gift of Dr. Stefanie Vogel, University of Maryland), (b) stimulated with LPS together with NECA (1 *μ*M), (c) stimulated with NECA alone (1 *μ*M), and (d) left unstimulated as a control group. Macrophages in each group were treated for 3, 6, 12, and 24 hours, and then total RNA was isolated and harvested.

### 2.2. Isolation of RNA

The cells from each plate were scraped in TRIzol Reagent (Invitrogen), and the RNA was isolated using the Zymo Research Corporation's isolation procedure. Ethanol (100%) was first added in a 1 : 1 volume ratio to the homogenate samples in TRIzol and vortexed. The sample was then loaded onto Zymo-Spin™ IIC Columns and treated with DNase I cocktail to remove DNA from the column. The columns were washed (2x) with Direct-zol™ followed by RNA Wash Buffer and the flow-through discarded. The Zymo-Spin IIC Column was then transferred to an RNase-free tube, and 50 *μ*l of DNase/RNase free water was added to elute the RNA, which was then stored at −80°C.

### 2.3. Reverse Transcription and Quantitative Real-Time PCR (Q-RT-PCR)

The RNA concentration for each sample was determined using a NanoDrop 2000c spectrophotometer (Thermo Fisher Scientific, Waltham, MA). For cDNA preparation, reverse transcription was performed using TaqMan Reverse Transcription Reagents (Applied Biosystems/Life Technologies, Grand Island, NY), and all incubations were performed in a C1000 Thermo Cycler (Bio-Rad, Hercules, CA). Q-RT-PCR reactions were set up to determine the expression of MYST 1, 2, 3, 4, and 5 RNA at each time point in the differently treated cells. A 96-well plate obtained from Applied Biosystems/Life Technologies (Grand Island, NY) was prepared for each MYST gene and for cyclophilin-D as an endogenous control. Each experiment was performed 3 times, and the reactions were in duplicate for each sample. For the gene of interest, the TaqMan probe and primer mixture were diluted to a 1 : 20 ratio. The TaqMan probes and primers used in these experiments were purchased from Applied Biosystems and are shown in Table 2. To each reaction, the following components were added: 1 *μ*l of 20x TaqMan probe and primer assay mixture, 10 *μ*l of 2x TaqMan Universal PCR Master Mix (both from Applied Biosystems), cDNA template (5 *μ*l), and water for a final volume of 20 *μ*l. Additionally, a control with no template was included in the experiment. Q-RT-PCR reactions were performed using an ABI 7000 Real Time PCR Thermocycler. Fold expression was normalized to that of unstimulated macrophages using the ΔΔCt method. All results were also normalized to the expression of cyclophilin-D, shown in previous studies to be constitutively expressed and minimally regulated by LPS.

### 2.4. Western Blot Analysis

Macrophages were lysed by adding the radioimmunoprecipitation assay (RIPA) buffer containing complete protease inhibitor cocktail (539134, Calbiochem, Billerica, MA). Samples were centrifuged at 10,000*g* for 10 minutes, and an aliquot was used for a Bradford-based protein determination. Cell lysates were boiled for 5 minutes with SDS-Laemmli buffer, and aliquots containing 50 *μ*g of protein were loaded onto 7.5% SDS-polyacrylamide gels for electrophoresis. Following electrophoresis, proteins were transferred to nitrocellulose membranes (Protran, Whatman, Dassel, Germany) using a Bio-Rad wet transfer system, according to the manufacturer's instructions. The membranes were then blocked with 5% low-fat milk in Tris-buffered saline with 0.1% Tween 20 for 1 hour, washed, and then incubated overnight at 4°C with an anti-Kat6b primary antibody (Novus Biologicals, Littleton, CO), or with an anti-nucleophosmin (NPM) primary antibody (Abcam, Cambridge, MA). The blots were then washed with Tris-buffered saline containing 0.1% Tween 20 and incubated for 1 hour with HRP-conjugated secondary IgG. Immunoreactive bands were developed using a chemiluminescent substrate, ECL Plus (GE Healthcare, Piscataway, NJ), and protein bands were detected by using a FluorChem analyzer (San Jose, CA).

### 2.5. Analysis of the Role of miRNA-487b in the Regulation of Kat6b Expression

**Figure 1:**
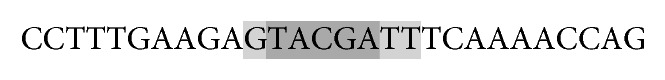
Region of the Kat6b 3′UTR sequence containing the putative miR-487b conserved binding site, indicated in bold. The complete 3′UTR (1995 bases) was inserted into the pLightswitch 3′UTR luciferase reporter plasmid, using the NHE1/XHO1 restriction sites, generating the pKat6bLuc-3′UTR plasmid (SwitchGear Genomics, Carlsbad, CA, Product ID S810637). Five bases (TACGA) were deleted from the miR-487b binding site to generate the mutant clone (pKat6bLucΔ3′UTR).

To determine the role of miR-487b in the regulation of Kat6b expression, two approaches were used. The first used the 3′UTR of the Kat6b gene cloned downstream of luciferase in a reporter plasmid, transfected into the RAW264.7 macrophage cell line. This 3′UTR contains a putative miR-487b binding site (Figure 1). A mutated 3′UTR lacking this miR-487b binding site was also tested (Figure 1). The second approach studied the effects of synthetic miR-487b mimics cotransfected into RAW264.7 cells with 3′UTR reporter plasmids, to determine the effects of miR-487b overexpression.

To determine whether miR-487b targets the expression of Kat6b through an effect on the putative miR-487b target site in the 3′UTR of the Kat6b gene, RAW264.7 cells were transiently transfected with one of the following pLightswitch 3′UTR luciferase reporter plasmid clones (SwitchGear Genomics, Carlsbad, CA): (a) pKat6bLuc-3′UTR, which contains the 3′UTR of the Kat6b gene cloned downstream of the luciferase gene in the pLightswitch-3′UTR plasmid, and also contains the RPL10 promoter 5′ of the luciferase insert. The RPL10 promoter is a constitutive promoter that is only minimally affected by LPS; (b) pKat6bLucΔ3′UTR, which contains the Kat6b 3′UTR with a specific deletion of the putative miR-487b binding site (Figure 1); (c) pEmptyLuc, which is the pLightswitch plasmid without a 3′UTR insert; and (d) pGAPDHLuc-3′UTR, which is a control construct with the GAPDH wild-type 3′UTR cloned downstream of luciferase. Expression of luciferase from this plasmid is unaffected by LPS treatment.

To determine whether the 3′UTRs were affected by the various treatments, RAW264.7 cells were transfected with 5 *μ*g of each plasmid using LipoD (SignaGen Laboratories, MD) according to the manufacturer's protocol for 18 hours. The cells were then replated in six-well plates (0.5 × 10^6^ cells in 1.5 ml of RPMI-10% FBS) and incubated at 37°C in the 5% CO_2_ tissue culture incubator for 24 hours. The medium was changed to RPMI 1640-1% FBS, and the cells were then stimulated with LPS (100 ng/ml), NECA (1 mM), and LPS/NECA or were left untreated. The plates were then incubated at 37°C for 6 hours, and the cells were then washed with PBS, lysed using Passive Lysis Buffer (Promega, Madison, WI), and assayed for firefly luciferase activity for each transfection. Changes in luciferase activity were determined by comparing the normalized luciferase activities of each of the test luciferase reporter constructs that are treated versus untreated transfection. Each treatment group was performed in triplicate, and each experiment was performed in duplicate.

The effects of a synthetic mimic (Qiagen, syn-mmu-miR-487b-3p miScript miRNA mimic, cat. number MSY0003184) on the expression of luciferase from the Kat6b 3′UTR plasmids alone and in response to LPS, NECA, and LPS/NECA were examined. A miR-433 mimic was used as a nonspecific control. The miR-487b and 433 mimics were transfected into RAW264.7 cells for 6 hours at a final concentration of 50 nM using HiPerFect (Qiagen), as described by the manufacturer. Following mimic delivery, 5 *μ*g of either pKat6bLuc-3′UTR, pKat6bMYST4Δ 3′UTR, or pEmptyLuc vectors was transfected into the RAW264.7 cells using LipoD (SignaGen Laboratories, MD), and the cells were processed and treated with LPS, NECA, and LPS/NECA as described above.

### 2.6. Statistical Analysis

Statistical analysis was performed with the unpaired Student *t*-test or analysis of variance followed by Tukey multiple comparison test. A *p* value < 0.05 was used to indicate statistical significance.

## 3. Results

### 3.1. miR-487b Expression Is Regulated in Murine Macrophages by LPS and LPS/NECA

In prior studies of the response to LPS (M1 activation) and LPSA/NECA (M2d activation), mi-RNA profiling analysis demonstrated a subgroup of miRs that were differentially regulated in response to LPS and LPS/NECA. These miRs included miR-877, miR-377-5p, miR-546, and miR-494, which were upregulated by LPS/NECA in comparison to LPS or NECA alone, and miR-487b, miR-212, miR-220, and miR-712, which were downregulated by LPS/NECA in comparison to LPS or NECA alone (Table 1).

**Figure 2:**
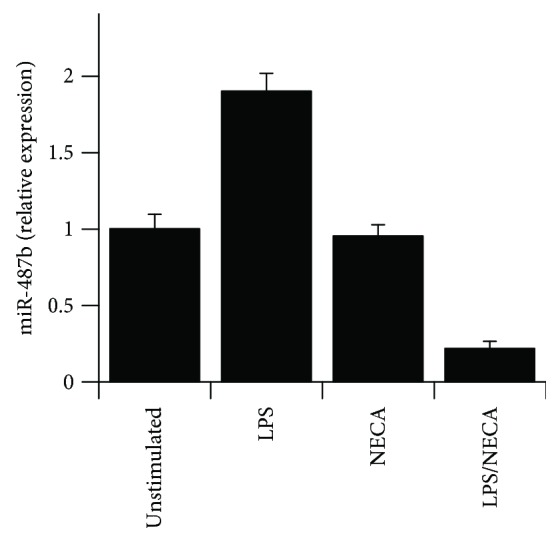
TaqMan Q-RT-PCR assays were performed using RNA samples from untreated macrophages or macrophages treated with LPS (100 ng/ml), NECA (1 *μ*M), or LPS (100 ng/ml) with NECA (1 *μ*M) for 12 hours. Samples from 3 separate experiments were each analyzed in duplicate, with data indicating mean fold change relative to untreated macrophages ± SE. Expression levels were normalized to the expression of cyclophilin-D as a housekeeping gene.

Q-RT-PCR analysis was used to confirm the regulation of miR-487b expression by LPS and LPS/NECA (Figure 2). LPS (100 ng/ml) upregulated miR-487b expression by ~2-fold. NECA alone had no effect, but NECA together with LPS strongly suppressed miR-487b expression in macrophages by ~80% in comparison to unstimulated macrophages. As miR-487b was upregulated by LPS (~2 fold) and strongly suppressed by LPS/NECA, we speculated that this miR might play a role in the switch of macrophages from an M1 to an M2d phenotype. Bioinformatic analysis of potential targets of miR-487b using TargetScan® and http://www.mirdb.org identified several genes with putative miR-487b target sites that are conserved across mammalian species. Kat6b was identified in these analyses as one of a small group of genes that are likely potential targets for miR-487b (Table 3).

### 3.2. Kat6b Expression Is Suppressed in Murine Macrophages by LPS

**Figure 3:**
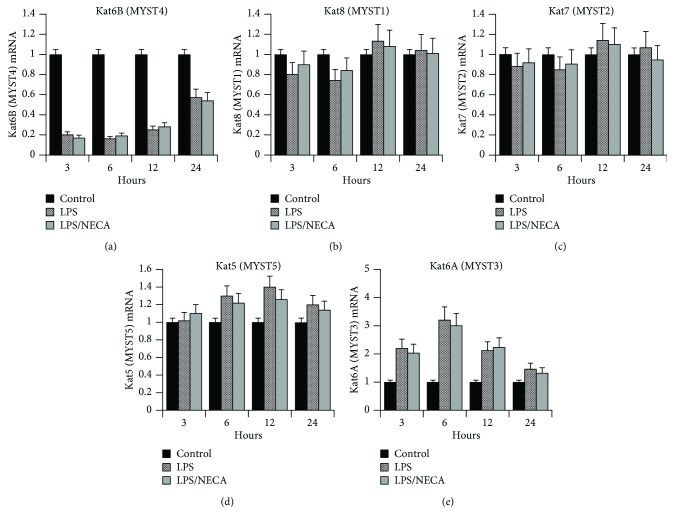
The expression of MYST family HATs (Kat6B, Kat6A, Kat5, Kat7, and Kat8) was determined using TaqMan Q-RT-PCR assays, with RNA samples from untreated macrophages or macrophages treated with LPS (100 ng/ml), NECA (1 *μ*M), or LPS (100 ng/ml) with NECA (1 *μ*M) for 3, 6, 12, and 24 hours. Data indicate mean fold change relative to untreated macrophages ± SE. Expression levels were normalized to that of cyclophilin-D as a housekeeping gene.

**Figure 4:**
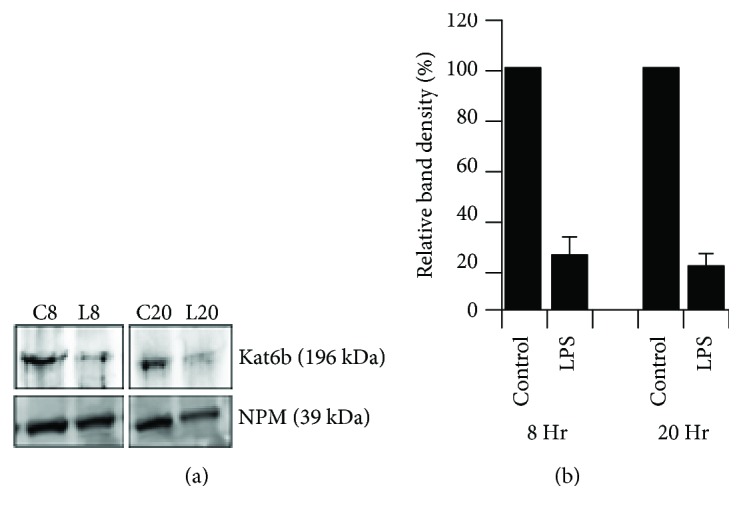
(a) Western blot analysis of the expression of Kat6B in untreated macrophages and macrophages treated with LPS (100 ng/ml) for 8 or 20 hours. Kat6B expression was compared to that of nucleophosmin (NPM), a housekeeping gene that is unaffected by LPS treatment. Samples were analyzed using 3 independent cell extracts, and typical Western blot images are shown. (b) Western blots were scanned and quantitated. Results show means ± SE (*n* = 3).

Kat6b is a histone acetyl transferase (HAT) that plays an important role in modifying histones and transcription factors and thus is involved in regulating gene expression. We initially analyzed the effect of LPS and LPS/NECA on the expression of Kat6b by macrophages in response to LPS using Q-RT-PCR. The results of this analysis are shown in Figure 3(a). Kat6b mRNA was strongly and rapidly suppressed (>80%) by LPS treatment of murine macrophages within 3 hours of treatment. This suppression was maintained through 12 hours following LPS treatment, with Kat6b mRNA levels still showing ~50% suppression by 24 hours. As shown in Figure 3(a), treatment with NECA, an adenosine A_2A_ and A_2B_ receptor agonist, did not significantly change this expression pattern. In addition to suppressing Kat6b mRNA, LPS also suppressed Kat6b protein expression.  Figure 4 shows a Western blot of Kat6b protein expression in macrophages after 8 and 20 hours of treatment with or without LPS. Nucleophosmin (NPM) was used as a housekeeping gene whose expression is not altered in response to LPS, and Kat6b levels were normalized to NPM expression. Kat6b protein expression was clearly suppressed in the LPS-treated macrophages. Epigenetic modulation of chromatin structure through regulation of histone demethylases and histone acetyl deacetylases (HDACs) has been implicated previously in classical (M1) and alternative (M2) pathways of macrophage activation. The potent suppression of the HAT Kat6b by LPS observed here suggests the potential importance of this gene in the regulation of macrophage phenotype.

### 3.3. Differential Regulation of MYST Family Genes in Murine Macrophages in Response to LPS

As expression of the HAT Kat6b was strongly suppressed in response to LPS, we investigated the expression of additional members of the MYST family of genes (MYSTs 1–5) to determine the specificity of this LPS-mediated suppression of Kat6b. All members of this family contain a MYST region of about 240 amino acids with a canonical acetyl-CoA-binding site and a C2HC-type zinc finger motif. Table 2 summarizes the nomenclature of the MYST family genes and the Q-RT-PCR primer/probes used to analyze their expression. The results of this analysis are shown in Figures 3(b)–3(e). MYST1 mRNA was not significantly regulated by LPS treatment (Figure 3(b)). Similarly, the levels of MYST2 (Figure 3(c)) and MYST5 (Figure 3(d)) mRNAs remained fairly constant through 24 hours following LPS treatment. In contrast, MYST3 mRNA levels were significantly elevated at 3, 6, and 12 hours following LPS treatment, with a 3-4-fold increase in expression, returning to baseline by 24 hours following LPS stimulation (Figure 3(e)). Treatment of macrophages with NECA alone did not affect the expression of any MYST gene. Also, treatment of macrophages with LPS together with NECA (LPS/NECA) did not alter the LPS-induced modulation of MYST gene expression. It thus appears that Kat6b and MYST3 are reciprocally regulated in response to LPS, with Kat6b being strongly suppressed, while MYST3 is induced.

### 3.4. Role of the Putative miR-487b Binding Site in the Kat6b 3′UTR in the Regulation of Kat6b Expression

**Figure 5:**
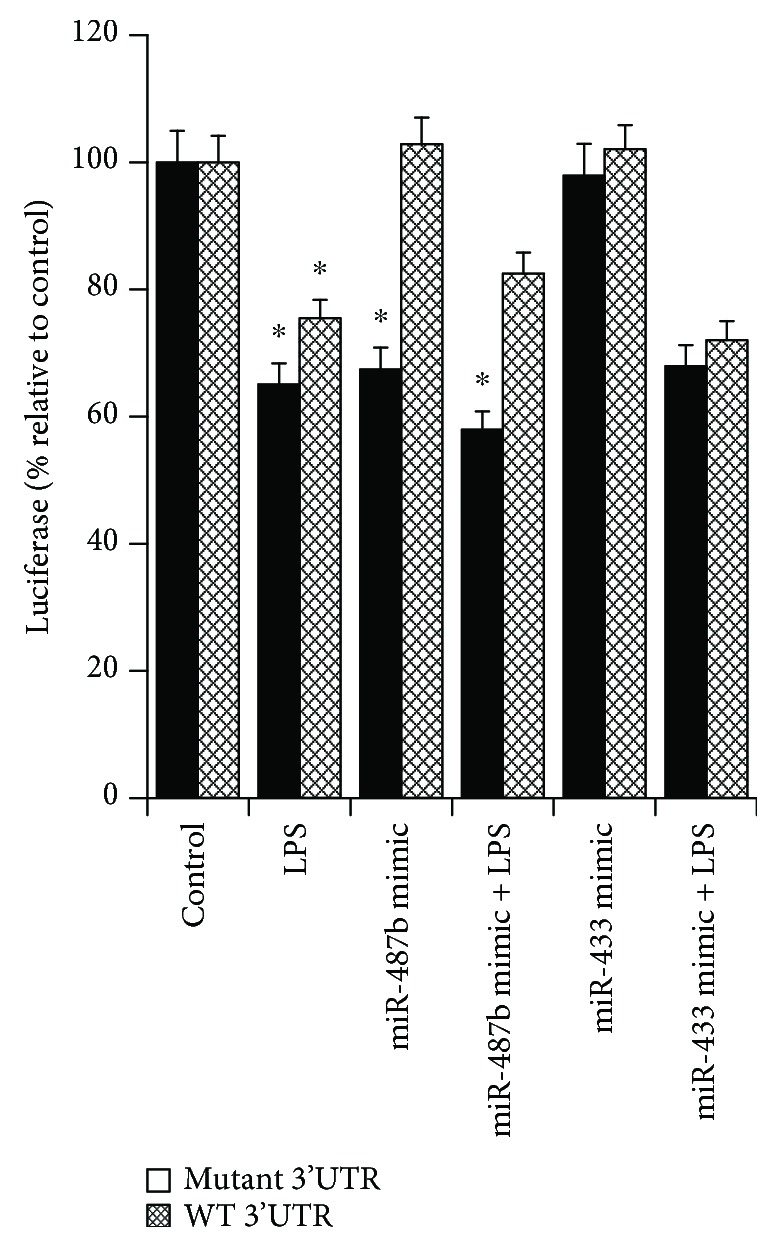
Analysis of the role of the miR-487b binding site in the 3′UTR of the Kat6b mRNA. The Kat6b 3′UTR was cloned downstream of the luciferase open reading frame in the pLightswitch-3′UTR reporter plasmid (pKat6bLuc-3′UTR). A second plasmid was prepared with the miR-487b binding site deleted (pKat6bLucΔ3′UTR). RAW264.7 macrophages were transfected with either pKat6bLuc-3′UTR or pKat6bLucΔ3′UTR. Transfected cells were then treated with LPS for 6 hr (100 ng/ml) and analyzed for luciferase expression (*n* = 3). RAW264.7 cells were also cotransfected with either pKat6bLuc-3′UTR or pKat6bLucΔ3′UTR together with either a synthetic miR-487b mimic or a nonspecific miR mimic (miR-433). The cells were then treated with LPS (100 ng/ml for 6 hr and analyzed for luciferase expression (*n* = 3). Data represent the mean ± SE. ^∗^ indicates samples with luciferase expression significantly different from control luciferase expression (*p* < 0.05).

To determine the role of the putative miR-487b binding site in the Kat6b 3′UTR in regulating Kat6b expression in response to LPS, we cloned the Kat6b 3′UTR into the pLightswitch-3′UTR plasmid downstream of the luciferase open reading frame, to generate the pKat6bLuc-3′UTR reporter plasmid. A second plasmid with the miR-487b binding site deleted, designated as pKat6bLucΔ3′UTR, was also engineered. These plasmids were transfected into RAW264.7 macrophages, which were then treated with LPS and examined for luciferase activity. As shown in Figure 5, LPS stimulation of RAW264.7 cells transfected with pKat6bLuc-3′UTR induced a ~35% decrease in luciferase activity, while in cells transfected with pKat6bLucΔ3′UTR, LPS induced a ~24% decrease in luciferase activity (*p* < 0.05). This suggests that the 3′UTR containing the miR-487b binding site plays a minor role in the suppression of the LPS-induced expression of luciferase, as the loss of the miR-487b binding site results in only a small increase in luciferase expression in response to LPS. This indicates that other factors in addition to miR-487b must be involved, as LPS still induces significant suppression in the absence of the miR-487b binding site.

To determine the effect of overexpression of miR-487b on the expression of luciferase from the wild-type and mutant reporter plasmids in the presence or absence of LPS, RAW264.7 macrophages were cotransfected with pKat6bLuc-3′UTR or pKat6bLucΔ3′UTR together with a synthetic miR-487b mimic or a nonspecific miR mimic (miR-433). As shown in Figure 5, the synthetic miR-487b mimic markedly suppressed luciferase activity from the wild-type vector (~35% suppression), while the nonspecific mimic had little effect. The effect of the miR-487b mimic was lost in the mutant vector. These results support the potential role of miR-487b to regulate Kat6b expression through binding to its binding site in the Kat6b 3′UTR. However, as is also shown in Figure 5, LPS still induced suppression of luciferase from the mutant vector, and this was not significantly affected by the miR-487b mimic. This finding suggests that while miR-487b can play a role in regulating expression, factors other than miR-487b must also play a role in the LPS-induced suppression.

## 4. Discussion

Macrophages play key roles in inflammation, wound healing, angiogenesis, and immune responses. Resting macrophages regulate the maintenance of tissue integrity but, in response to inflammatory stimuli, change their gene expression profile to produce inflammatory or anti-inflammatory cytokines, growth, and angiogenic factors. The rapid and profound changes in the expression of genes in macrophages are mediated at several levels, including transcriptional control, as well as posttranscriptional regulation of translation and mRNA and protein stability.

Classical (“M1”) activation of macrophages is induced by IFN-*γ* and/or TLR agonists such as LPS (TLR4 agonist) and is characterized by the rapid and transient induction of inflammatory cytokines such as TNF*α* and IL-12 and NOS-3 (iNOS). In contrast, alternative activation pathways that induce an anti-inflammatory phenotype have been described. These have generally been termed “M2” macrophages [11, 39]. Activation by IL-4, for example, induces an anti-inflammatory phenotype termed M2a, characterized by low expression of inflammatory cytokines and elevated expression of the anti-inflammatory cytokine IL-10, IL-1R*α*, as well as markers such as CD206 (MR), CD163, MHCII, Ym1, FIZZ-1, and arginase-1 [39–41]. We have previously described an “alternatively activated” macrophage phenotype that we have termed “M2d” [15, 16, 42]. This phenotype is induced by TLR2, 4, 7, and 9 agonists in a MyD88-dependent manner, in synergy with agonists of adenosine A2A and A2B receptors [15, 18–20]. M2d macrophages express low levels of inflammatory cytokines and high levels of IL-10 and the angiogenic growth factor VEGF. Induction of this phenotype involves transcriptional upregulation of HIF1*α* and posttranscriptional suppression of phospholipase-C*β*2 (PLC*β*2) [42, 43].

To determine the mechanism of PLC*β*2 suppression in response to M2a activation, we performed a global screening of miRNAs expressed in response to LPS, to NECA (an AR agonist), and to LPS together with NECA (M2d activation conditions). MiRNAs that are regulated by LPS have been published in prior studies [24, 33, 36, 44–47]. In the current study, miRNAs specifically modulated by LPS with NECA versus LPS alone were identified. As shown in Table 3, a subgroup of miRNAs was either up- or downregulated in response to LPS/NECA versus LPS alone. We confirmed the changes in expression of miR-487b, which was found to be mildly induced by LPS, but strongly suppressed by LPS with NECA (Table 3 and Figure 2). Bioinformatic analysis of potential targets of miR-487b using TargetScan and http://www.mirdb.org identified a group of genes with putative miR-487b target sites conserved across mammalian species (Table 3). The HAT Kat6b was identified in these analyses as one of a small group of genes that are potential targets for miR-487b.

There has been much interest recently in the role of epigenetic modulators in the regulation of macrophage activation pathways [48–53]. In particular, chromatin remodeling induced by targeted epigenetic modifications such as histone methylation or demethylation, as well as acetylation or deacetylation, may lead to gene activation or repression [40, 54]. Histone deacetylases (HDACs) have been shown to play an important role in macrophage M1 and M2 activation; however, the role of histone acetyl transferases (HATs) in regulating macrophage activation has received little attention. HATs and HDACs are families of enzymes that modulate chromatin structure, thus affecting inflammatory gene expression [55, 56]. Mice lacking HDAC3 display a polarization phenotype similar to IL-4 induced alternative activation and are hyperresponsive to IL-4 stimulation, suggesting that HDAC3 is an epigenomic brake in macrophage M2a activation [53, 57–59]. By extension, this would suggest that HATs might provide a stimulus to M2 activation, in contrast to the effects of HDACs. However, the role of HATs in regulating macrophage M1/M2 polarization remains to be determined.

HAT complexes of the MYST family are named after the four founding family members, MOZ, Ybf2 (Sas3), Sas3, and Tip60 [60, 61]. Other members of this family include Esa1, MOF, MORF, MSL, and HBO1 (Table 2). MYST family HATs are typically characterized by the presence of zinc fingers and chromodomains and are involved in acetylation of lysine residues on histones H2A, H3, and H4 [62–64]. As the HAT Kat6b was identified in this study as a potential target of miR-487b, we examined the effects of LPS on the expression of Kat6b and also of the other members of the Kat family of HATs (Kat6a, Kat5, Kat7, and Kat8). Kat6a and Kat6B form stable multisubunit complexes, MOZ and MORF, respectively [65]. The MOZ/MORF complex is responsible for acetylation of a substantial portion of histone H3, and possibly of other histones. The HAT activity of the MOZ/MORF complex is required for normal development, including hematopoiesis and skeletogenesis. Mutations of Kat6B have been identified in patients with Say-Barber-Biesecker syndrome and with genitopatellar syndrome [66–68]. In a form of acute myeloid leukemia, there is a translocation of the N-terminal portion of Kat6b in frame with CBP [62]. A translocation resulting in fusion to TAFII also leads to acute myeloid leukemia [63]. Disruption of Kat6b also leads to a Noonan syndrome-like phenotype and hyperactivated MAPK signaling in both humans and mice [69]. Mutant mice deficient in Kat6b are reported to develop poorly, exhibiting growth retardation, facial dysmorphism, skeletal abnormalities, and developmental brain anomalies, leading to their designation as “Querkopf” (“Strange head”) mice [69]. No studies on the inflammatory and immunological responses of these mice have been reported to date.

Since Kat6B (MYST4, MORF) was identified as a potential target of miR-487b (Figure 1), we studied the expression of Kat6b, as well as the other members of the MYST family of HATs, in the response of macrophages to LPS (M1) and LPS/NECA (M2d) activation. As shown in Figure 3, LPS induced a rapid, strong, and sustained suppression of Kat6b mRNA expression. Strong suppression (>80%) was observed by 3 hours following LPS treatment and sustained through at least 12 hours. After 24 hours, 50% suppression was still apparent. In contrast, Kat6A (MYST5, MOZ) expression was stimulated by LPS and showed a reciprocal pattern of expression to that of Kat6A. The other members of the MYST family (Kats 5, 7, and 8) were not affected by LPS treatment. Surprisingly, the expression patterns of Kat6A and Kat6B in response to LPS/NECA were the same as those with LPS alone, despite the fact that miR-487b expression is strongly suppressed by LPS. We propose in the light of published literature implicating HDACs in M2 activation [53, 57–59] that the strong downregulation of the HAT Kat6b induced by LPS may play a reciprocal role with HDACs in regulating macrophage polarization. We are currently testing this hypothesis.

To determine the role of miR-487b in the regulation of Kat6B suppression by LPS in macrophages, we cloned the intact Kat6B 3′UTR and a mutated 3′UTR lacking the miR-487b core binding sequence into a luciferase reporter plasmid. These plasmids were transfected into the macrophage cell line RAW264.7 either alone or together with a miR-487b mimic. LPS suppressed luciferase expression in the intact plasmid, and this suppression was only mildly abrogated by loss of the miR-487b binding site; however, the synthetic miR-487b mimic markedly suppressed luciferase activity from the wild-type vector, while the nonspecific mimic had little effect (Figure 5). The suppressive effect of the miR-487b mimic was lost in the mutant vector. Together, these results suggest that while the miR-487b site in the Kat6B 3′UTR plays a role in the LPS-mediated suppression of Kat6B, other factors in addition to miR-487b must also contribute. As LPS/NECA strongly suppresses miR-487b expression in comparison to LPS alone, the lack of effect of LPS/NECA on the expression of Kat6B also clearly suggests that miR-487b is not the sole factor involved in the suppression of Kat6B by LPS. In this context, it is of interest to note that miR-487b was recently implicated as a negative regulator of bone marrow-derived macrophage activation by targeting IL-33 production. miR-487b suppressed IL-33 production during the differentiation of bone marrow-derived macrophages by binding to the 3′UTR of IL-13 mRNA and suppressing its translation [70]. Nevertheless, the role of miR-487b modulation of macrophage M1/M2 polarization remains unclear.

In summary, we have found that the HAT Kat6B is strongly suppressed in macrophages by LPS (M1 activation), while Kat6A is reciprocally upregulated. This pattern is not altered by LPS/NECA (M2d activation), despite the observation that miR-487b, a putative regulator of Kat6B expression, is strongly suppressed by LPS/NECA. As Kat6B is a HAT that has the potential to modulate gene expression by its effects on chromatin accessibility, we are continuing our studies into the potential roles of this epigenetic modulator in macrophage activation pathways.

## Figures and Tables

**Table 1:** MiRNAs differentially regulated in murine peritoneal macrophage by LPS (M1 activation) versus LPS/NECA (M2d activation).

miRNA	Regulation by LPS (fold change in comparison to untreated control macrophages)	Regulation by LPS/NECA (fold change in comparison to LPS treated macrophages)
		*Fold increase*
Mmu-miR-483	1.3	8.8
Mmu-miR-877	1.4	6.5
Mmu-miR-337-5p	1.3	5.3
Mmu-miR-546	1.0	5.2
Mmu-miR-494	1.1	4.7
Mmu-miR-615-3p	1.5	3.5
		*Fold Decrease*
Mmu-miR-770-5p	1.1	−15.6
Mmu-miR-487b	1.9	−8.7
Mmu-miR-220	1.3	−7.3
Mmu-miR-212	1.0	−7
Mmu-miR-712	1.0	−5.8
Mmu-miR-715	1.9	−4.6
Mmu-miR-204	1.5	−3.9
Mmu-miR-211	1.5	−3.8
Mmu-miR-296-5p	1.9	−3.7
Mmu-miR-295	3.7	−3.6

**Table 2:** 

MYST family members (with alternative names)	TaqMan primer/probe serial number
MYST1 (Kat8, MOF)	Mm00458911_m1
MYST2 (Kat7, ORC1, and HBO1)	Mm00624391_m1
MYST3 (Kat6A, MOZ, ZNF220, and RUNXBP2	Mm01211941_m1
MYST4 (Kat6b, MORF, and Querkopf)	Mm00450564_m1
MYST5 (Kat5, TIP60, and HTATIP)	Mm01231512_m1

**Table 3:** Transcripts with conserved 3′UTR sites for miR-487b.

Gene name	Gene symbol	Conserved sites	8-mer	7-mer
Chemokine Z (C-X-C motif) ligand 12	CXC 12	1	1	
NOTCH-regulated ankyrin repeat protein	NRARP	1	1	
Astrotactin-1	ASTN1	1		1
Nasal embryonic LHRH factor	NELF	1	1	
K(Lysine) acetyl transferase 6B	KAT6B	1		1
Protein kinase C, alpha	PRKCA	1		1
EF-hand domain family, member D2	EFHD2	1		1
Mitogen-activated protein kinase kinase 4	MAP2K4	1		1
Zinc finger protein 219	ZNF219	1		1
Ring finger protein 165	RNF165	1		1
Protocadherin 7	PCDH7	1		1
EPH receptor A3	EPHA3	1		1
Insulin receptor substrate 1	IRS1	1		1
POU class 2 homeobox 1	POU2F1	1		1

## Data Availability

All relevant data is included in the manuscript. Additional requests for underlying data should be submitted to the corresponding author.
